# Continuous Glucose Monitoring in People at High Risk of Diabetes and Dysglycaemia: Transforming Early Risk Detection and Personalised Care

**DOI:** 10.3390/life15101579

**Published:** 2025-10-10

**Authors:** Alexandros L. Liarakos, Grigorios Panagiotou, Maria Chondronikola, Emma G. Wilmot

**Affiliations:** 1 Department of Diabetes and Endocrinology, University Hospitals of Derby and Burton NHS Foundation Trust, Royal Derby Hospital, Derby DE22 3DT, UK; alexandros.liarakos1@nhs.net; 2 School of Medicine, Faculty of Medicine and Health Sciences, University of Nottingham, Nottingham NG7 2UH, UK; 3 Institute of Metabolic Science, University of Cambridge, Cambridge CB2 0QQ, UK; gp558@cam.ac.uk (G.P.); mc2425@medschl.cam.ac.uk (M.C.)

**Keywords:** diabetes, continuous glucose monitoring, precision medicine, cardiovascular risk, prevention

## Abstract

Continuous glucose monitoring (CGM)-based interventions have been predominantly conducted in people with established diabetes. Recently, there has been an increasing interest in using CGM for clinical and research purposes in people without diabetes. In this review, we describe the current evidence regarding the use of CGM in people at high risk of diabetes. To date, there is no strong evidence to support the global implementation of CGM in individuals who are at risk of developing diabetes. However, there are promising results highlighting the benefits of CGM in specific populations such as people living with obesity, prediabetes, gestational diabetes mellitus, metabolic dysfunction-associated steatotic liver disease, other endocrinopathies, and genetic syndromes. Also, CGM has shown promising potential in people with positive islet autoantibodies and pre-symptomatic type 1 diabetes, those treated with medications that induce hyperglycaemia or diabetes, and individuals receiving solid organ transplantation who are at risk of post-transplant diabetes mellitus. However, larger studies are needed to confirm these preliminary results. CGM-derived data are not currently validated for the diagnosis of diabetes. There is no CGM-derived definition of normoglycaemia in people without diabetes. Looking to the future, CGM metrics, in tandem with physical activity, dietary intake, and clinical parameters, and eventually bioinformatics, may inform personalised risk scores for precision prevention of individuals at risk. We conclude that further research is needed to clarify the indications, drawbacks, and feasibility of CGM use in people at high risk of diabetes to identify those groups who could benefit most from this technology.

## 1. Introduction

Continuous glucose monitoring (CGM) is a minimally invasive technology that allows for measurements of interstitial fluid glucose concentration. CGM provides an alternative to standard monitoring methods of glucose control, such as self-monitoring and capillary blood glucose (CBG) measurements with finger-prick testing. While finger-prick testing can be painful and inconvenient for some individuals, CGM has revolutionised the care for people living with both type 1 and type 2 diabetes mellitus (T2DM), improving glycaemia, hypoglycaemia, diabetic ketoacidosis (DKA), and hospital admissions [1,2,3,4].

**Figure 1:**
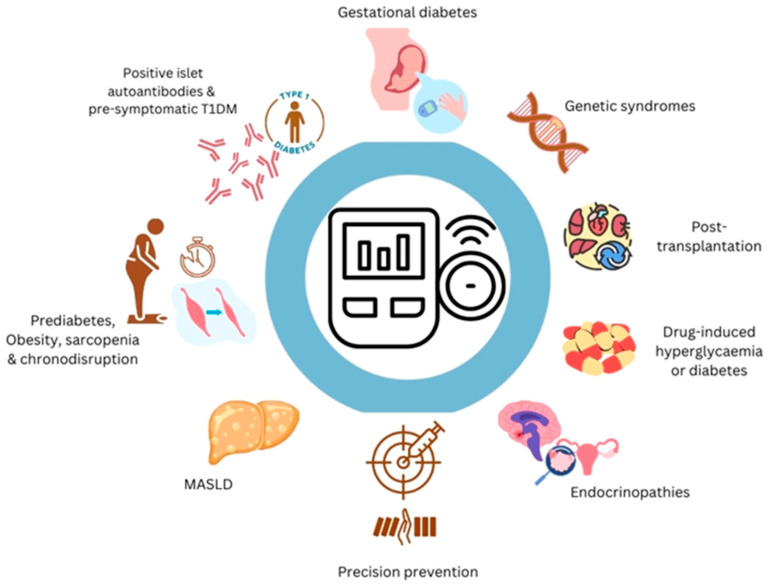
Potential applications of continuous glucose monitoring in people at high risk of diabetes. Abbreviations: T1DM, type 1 diabetes mellitus; MASLD, metabolic dysfunction-associated steatotic liver disease.

Although CGM-based interventions have been predominantly conducted in people with established diabetes [5], in recent years, the role of CGM as a potential preventive and/or predictive tool, before diabetes is clinically manifested, is being explored [6]. CGM could be a significant consideration for people who have a high risk of developing diabetes (Figure 1).

The aim of this narrative review is to evaluate the current evidence around the use of CGM in people at high risk of diabetes, based on randomised controlled trials (RCTs), observational studies, systematic reviews, and meta-analyses. In contrast to previous reviews that focused on individual subgroups or wellness applications, this review integrates evidence across multiple high-risk populations and explores the potential of CGM in precision prevention and real-time biofeedback with or without bioinformatics-driven risk stratification. We used the keywords ‘type 1 diabetes’, ‘type 2 diabetes’, and terms related to high risk of diabetes alone and in combination to retrieve the available literature data from PubMed from inception until April 2025. High-risk groups included people with pre-symptomatic type 1 diabetes with positive islet autoantibodies, pre-type 2 diabetes or other metabolic dysfunction associated with changes in body composition, metabolic dysfunction-associated steatotic liver disease (MASLD), and gestational diabetes mellitus (GDM), as well as other endocrinopathies associated with glucose intolerance, including polycystic ovarian syndrome (PCOS) and acromegaly. They may also include individuals with solid organ transplantation, those treated with specific medications such as steroids and immune checkpoint inhibitors, and people with specific genetic syndromes affecting glucose and/or insulin metabolism. Herein, dysglycaemia is used as a general term for elevated blood glucose, including impaired fasting glucose, impaired glucose tolerance, prediabetes, or type 2 diabetes. Prediabetes refers to impaired fasting glucose and/or impaired glucose tolerance as per American Diabetes Association/World Health Organisation criteria [7]. Early T2DM refers to a diagnosis of type 2 diabetes within the last 5 years or newly diagnosed at screening. Given the heterogeneity of populations and study designs, a systematic review or meta-analysis was not feasible. Hence, we adopted a narrative approach to synthesise the available evidence. Table 1 summarises the potential future applications and limitations of CGM across high-risk groups for dysglycaemia and diabetes.

## 2. Positive Islet Autoantibodies and Pre-Symptomatic Type 1 Diabetes

Type 1 diabetes mellitus (T1DM) is an autoimmune disease with a continuum of stages ranging from genetic risk to the development of metabolic disease. Stage 1 T1DM is defined by islet autoantibody positivity in a person with normoglycaemia. Stage 2 is characterised by glucose intolerance or dysglycaemia, while stage 3 T1DM is characterised by the biochemical criteria for diabetes in asymptomatic (stage 3a) or symptomatic people (stage 3b) who will require insulin therapy [18]. In children, stage 1 T1DM is associated with an 85% risk of progression to stage 3b T1DM within 15 years [19]. Although the data in adults are limited, current evidence suggests that many adults with multiple positive islet autoantibodies and early-stage T1DM can still develop clinical T1DM, although the rate of progression is slower than in children [20]. Therefore, the detection of glycaemic deterioration at an earlier stage in pre-symptomatic T1DM is crucial to identify those who may benefit most from early interventions to delay or prevent the progression to symptomatic T1DM, such as immunotherapy [21].

The oral glucose tolerance test (OGTT) and haemoglobin A1c (HbA1c) are the standard metrics for staging and risk prediction for the development of symptomatic T1DM. Recently, CGM has been utilised in individuals with pre-symptomatic T1DM to identify early glycaemic abnormalities and predict the risk of progression to clinical T1DM [8,22]. Ongoing research assesses the role of blinded CGM in identifying individuals with positive diabetes antibodies, even those with normal OGTT, who are likely to progress to stage 3 T1DM [8,23]. Small studies using CGM in children and youth with stage 1 or stage 2 T1DM indicated that glucose ≥ 7.8 mmol/L (≥140 mg/dL) for >10% per day is associated with an 80% risk of progression to T1DM within 12 months [8]. A longitudinal analysis of 34 multiple autoantibody-positive participants (baseline median age 16.6 years) showed that repeated 5-day CGM and HbA1c measurements were almost as effective as OGTT in predicting stage 3 T1DM [24], although this was not supported by a different study [25]. CGM has the potential to offer a more convenient option for long-term clinical monitoring and can provide insight into factors impacting glycaemia in the real-world environment, including assessments of overnight glycaemic variability. Indeed, it was more acceptable to children and their families compared with HbA1c or OGTT [8,9].

Regarding specific CGM metrics, two studies indicated that compared with non-progressors, progressors had significantly higher parameters or glycaemic variability [8,23]. Specifically, the cut-off of 20 mg/dL (1.1 mmol/L) standard deviation (SD) had 81% specificity and sensitivity for diabetes prediction, and for the cut-off of 37 mg/dL (2.1 mmol/L), the mean amplitude of glucose excursion had 69% sensitivity and 91% specificity for diabetes prediction. 10% time > 140 mg/dL (7.8 mmol/L) had 91% specificity and 88% sensitivity for diabetes prediction [8].

A recently published consensus guidance has included CGM as a monitoring method for individuals with islet autoantibody-positive pre-stage 3 T1DM (12). However, it should be noted that the use of CGM-derived criterion (CGM values >7.8 mmol/L [>140 mg/dL] for 10% of time over 10 days’ continuous wear for stage 2 T1DM with ≥2 autoantibodies) did not achieve consensus and CGM metrics are not part of the current American Diabetes Association or International Society for Paediatric and Adolescent Diabetes guidelines on staging criteria in T1DM [18,26]. The guidance [22] advises that in children with multiple islet autoantibodies, a 10–14-day CGM can be used intermittently to monitor glucose levels at a similar frequency as HbA1c measurement, while the sensors should ideally be blinded to the user and interpreted by trained healthcare professionals (HCPs). In adults with multiple positive autoantibodies and confirmed stage 2 T1DM, the guidance advises that glycaemia should be monitored using HbA1c every 6 months, combined with another monitoring method, including blinded CGM, which should be applied and interpreted by trained HCPs. However, it should be noted that these statements were GRADE (Grading of Recommendations, Assessment, Development, and Evaluations) ‘E’ for both children and adults, and thus should be interpreted with caution. In women with confirmed islet autoantibody positivity who become pregnant, the consensus panel advised for an OGTT, HbA1c, or CGM soon after pregnancy is confirmed [22].

However, there are aspects that need to be considered before scaling up the application of this technology, and currently, the use of CGM in this context is rather premature; further research is required to understand diagnostic thresholds, optimal monitoring frequencies, cost-effectiveness, and the impact of CGM on well-being in the context of pre-symptomatic type 1 diabetes.

## 3. Metabolic Dysfunction

*Prediabetes and altered body composition.* Early detection of prediabetes and/or early-stage T2DM allows for early intervention, including substantial weight loss aiming for prevention or remission [27,28,29]. It has been suggested that CGM could assist in the detection of prediabetes and/or early T2DM [30]. Current strategies for the diagnosis of prediabetes include fasting glucose, OGTT, and/or HbA1c measurements [31]. However, these methods vary in terms of their limitations, generalisability, and diagnostic accuracy. HbA1c remains the most used test; however, it may be unreliable in patients with haemoglobinopathies and/or other more common disorders, as well as a short duration of hyperglycaemia [32]. Relying on fasting glucose may miss postprandial hyperglycaemia, and OGTT is a clinic-based procedure requiring strict protocol adherence and repeated blood sampling; thus, it can be expensive and inconvenient to provide, which limits its widespread use. Contrariwise, CGM-based glucose variability (GV) is associated with reduced β-cell function in prediabetes [33] and has been proposed as a marker of early dysglycaemia [34,35]. CGM has been shown to have a potential role in early detection of dysglycaemia compared to fasting plasma glucose or HbA1c in people without diabetes [36]. However, in a different study, CGM-derived mathematical modelling produced similar sensitivity to HbA1c for the diagnosis of prediabetes and diabetes and significantly higher sensitivities compared to OGTT [37]. Therefore, larger prospective cohort studies are needed to fully explore the diagnostic potential of CGM in the early detection of dysglycaemia. However, it must be emphasised that at present, no study has confirmed CGM as a validated tool for the pre-diagnosis of type 2 diabetes, and conventional diagnostic tests remain the gold standard.

Central and visceral body fat accumulation strongly correlates with insulin resistance, a precursor to the development of T2DM [38]. Lifestyle and nutritional interventions remain at the core of prevention and management of obesity and its related complications [39], and more recently, a significant breakthrough has been made with weight-loss pharmacotherapy [40]. However, the role of novel technologies, such as CGM, in predicting or monitoring future risk for the development of diabetes in people living with obesity is less explored. In previous studies, CGM has been predominantly used to assess GV of research participants living with obesity, while its use in providing biofeedback to support adherence to weight loss interventions, encourage behavioural changes, or guide interventions manipulating the timing of dietary intake is less explored [41]. Indeed, it has been shown that self-monitoring with CGM improved adherence to lifestyle modifications [42] and integrating CGM in weight loss interventions could provide additional benefits, including sustained weight loss. CGM use in the Diabetes Prevention Program was feasible, acceptable, and motivational in making lifestyle and dietary changes in adults at high risk of diabetes or prediabetes [43], with similar results in adolescent populations [44]. To the best of our knowledge, CGM has not been used in Diabetes Remission Programmes for people with recently diagnosed T2DM.

Furthermore, age-related decline in muscle mass and function, termed sarcopenia, is associated with frailty, loss of independence, and increased mortality [45,46]. A reduction in muscle mass is also associated with postprandial hyperglycemia, due to reduced glucose clearance and/or lower suppression of endogenous glucose production, and impaired glucose tolerance (IGT) [47]; however, the latter requires a clinic-based OGTT with several blood draws at different timepoints to identify. Interestingly, two small studies recently showed that CGM-derived serial interstitial glucose concentrations following an OGTT could be used to identify muscle insulin resistance. This was assessed via a modified insulin-suppression test and expressed as steady-state plasma glucose in a community setting, thereby reducing the need for clinic-based OGTTs [48,49]. Thus, there could be potential for CGM use in individuals with suspected sarcopenia or sarcopenic obesity to detect patterns of postprandial IGT or muscle insulin resistance outside a hospital environment, subsequently triggering further investigations and directing appropriate management.

Another risk factor for prediabetes is chronodisruption. This disruption of normal circadian rhythmicity due to altered working patterns, including late-night work or shifts, is also associated with an increased risk of T2DM [50,51] and cardiometabolic diseases [52,53]. In the only published study examining the effects of shift work on CGM metrics, in a cohort of 450 male daytime and shift workers without diabetes with at least 5 years of altered working patterns, shift workers had a worse cardiometabolic profile and higher nighttime mean blood glucose, with higher glucose levels at 3 a.m., while other CGM metrics were essentially similar between study groups [54]. In the same study, obesity was an independent predictor of nighttime glycaemia [54], suggesting a role of nighttime glycaemia as an early sign of glucose dysmetabolism and highlighting the need for lifestyle guidance in individuals working unsocial hours. Regarding sleep metrics, sleep quality was previously studied in people with T1DM [55] and T2DM [56]; however, in a recent study with 40 healthy individuals, the authors showed that sleep latency is directly associated with CGM-assessed SD of GV [57].

*Gestational diabetes mellitus (GDM).* GDM is the most frequently occurring metabolic disorder in pregnancy, with a global prevalence of 14% [58], and it is associated with maternal and perinatal/neonatal adverse outcomes, including a long-term, increased risk of T2DM [59,60]. The gold standard for GDM diagnosis is via an OGTT performed between 24 and 28 weeks of gestation, and self-monitored blood glucose with finger-prick testing is the routine method for glucose monitoring.

CGM improves outcomes in pregnant women with pre-existing T1DM, both as a standalone option [61] and as part of a hybrid closed-loop system [62]. It is also recommended in insulin-treated non-T1DM populations with problematic, severe hypoglycaemia or labile glucose control for insulin titration and/or prevention of hypoglycaemia [63]. CGM may have potential in those with T2DM, and studies are underway to assess this [64]. CGM may also possibly support an earlier diagnosis of GDM, via monitoring and encouraging adherence to lifestyle recommendations, as well as inform a personalised risk assessment for disease progression or complications later in pregnancy [65]. Indeed, people with OGTT-confirmed GDM in the second trimester showed earlier signs of hyperglycaemia detected by CGM [66,67]. CGM in the first trimester performed better in predicting GDM [68] and/or negative pregnancy outcomes compared to standard risk assessment methods [12], supporting its potential as a screening and risk stratification tool, as early as 13–14 weeks of gestation. Nevertheless, these findings are exploratory, and CGM in early pregnancy cannot currently be recommended as a validated screening tool outside of research settings. At a later stage, CGM data between 26 and 32 gestational weeks were predictive of glucose-lowering treatment requirements after GDM diagnosis [69]. In different studies, commonly used [70] or diurnal, glucose rhythmicity data derived by CGM [10] were associated with both maternal and neonatal adverse outcomes, and CGM-based machine-learning models were predictive of large-for-gestational age offspring [11]. Also, CGM is a useful tool in the self-management of GDM and may empower women with GDM during their pregnancies [71].

However, while CGM applications in pregnancy are promising, current medical costs may limit wider use. In a recent RCT, finger-prick testing was less expensive; however, CGM was more beneficial for ideal gestational weight gain [72], suggesting a role of CGM in improving compliance with healthy behavioural choices aiming for weight maintenance during gestation. CGM metrics throughout gestation may also offer a better insight into the pathophysiology and trajectory of GV during pregnancy, thereby informing tailored strategies for GDM prevention [73]. For example, pregnant women with obesity but no diabetes had higher CGM day/night metrics compared to normal-weight individuals with similar fasting glucose and HbA1c levels [74]. Importantly, CGM appears to be a well-received technology in women with GDM [75] that can complement at-home OGTT assessment [76] to reduce clinic appointment times and improve patient satisfaction rates. However, the impact of CGM on foetal and maternal outcomes, as well as CGM targets, should be defined prior to widespread implementation. While pregnancy-specific TIR >70% is advised for pregnant women with T1DM, there is currently limited evidence about specific CGM targets in GDM or pregnant women with T2DM [77].

*Non-diabetic hypoglycaemia.* CGM has been used in people with obesity following bariatric surgery. While postprandial hypoglycaemia after bariatric surgery is a recognised side effect, CGM studies have also revealed higher rates of asymptomatic, nocturnal hypoglycaemic episodes [78]. CGM could detect distinctive postprandial glucose trends, including earlier and higher peaks, lower nadir levels, increased GV, and frequent episodes of asymptomatic, postprandial, and/or overnight hypoglycaemia [79]. In a meta-analysis of eight studies, hypoglycaemia rates were similar between Roux-en-Y gastric bypass (RYGB) and sleeve gastrectomy; however, RYGB was associated with higher glycaemic variability in CGM metrics, due to hormonal and glycaemic changes [80]. However, a prospective study comparing post-RYGB individuals with or without T2DM vs. non-operative controls (*n* = 7 per group) identified significant differences between groups, with individuals without diabetes exhibiting high GV and the longest duration of hypoglycaemia compared to people with diabetes, possibly due to improved insulin sensitivity and the preserved effect of endogenous incretins [81]. Conversely, Dorcely et al. found that people without T2DM had lower glycaemic variability than those with diabetes post-sleeve gastrectomy, in whom high glycaemic variability may persist after surgery [82]. Importantly, as post-bariatric surgery hypoglycaemia may not resolve for several months [83], CGM sensors could prevent severe hypoglycaemic episodes, especially in those with impaired awareness, through diet modification and proactive self-management [84].

Furthermore, it has been proposed that CGM could be used in the screening or monitoring of individuals with suspected insulinoma, with a coefficient of variation >19% being suggestive of endogenous hyperinsulinism compared to functional hypoglycaemia [85]. CGM was also helpful in the intraoperative management of those with insulinomas [86].

*Metabolic dysfunction-associated steatotic liver disease (MASLD).* While postprandial hyperglycaemia is associated with muscle insulin resistance, impaired fasting glucose represents liver-specific insulin resistance related to hepatic gluconeogenesis and glycogenolysis [47]. Despite the recent advancements in pharmacotherapy of MASLD [87], the main course of management currently lies with prevention of disease progression through lifestyle interventions and weight loss [88].

Dysglycaemia is a major contributor to the pathophysiology of MASLD [89,90] and CGM metrics, including time-in-range (TIR), estimated HbA1c, mean of daily differences (MODD), J_index, and high blood glucose index, have been correlated with both biochemical (e.g., aspartate transaminase/alanine transaminase ratio and gamma glutamyl transferase levels) and sonographic features (liver attenuation, viscosity, and sound speed) outcomes in a large study of people without diabetes [91]. Additionally, CGM-derived dysglycaemia was associated with the degree of fibrosis in adult [92] or paediatric [93] populations with MASLD, suggesting a role of CGM in risk prediction and management of such populations. Importantly, nighttime glycaemic indices, including TIR and MODD, which can only be obtained via CGM in an out-of-hospital setting, were the strongest predictors of liver steatosis [92]. While there is limited evidence on the role of CGM as a target for the prevention of disease progression prospectively, and/or as a monitoring tool in prescribed behavioural or pharmacological interventions, these results highlight the potential of CGM-derived data in individuals with MASLD. However, the associations between CGM metrics and liver steatosis/fibrosis are highly confounded by adiposity, dietary intake, sleep, and concomitant medications. Longitudinal cohorts incorporating serial imaging, biomarkers, and wearable-derived lifestyle data are required to establish directionality and the incremental predictive value of CGM metrics over traditional risk scores.

*Other endocrinopathies.* There is limited evidence regarding the potential CGM benefits in secondary diabetes related to hormone excess. In one study, women with polycystic ovarian syndrome (PCOS) showed delayed postprandial times to peak glucose and higher amplitude of postprandial glucose excursions compared to healthy controls [94]. Future larger cohorts are needed to prospectively analyse the role of diurnal GV using CGM in this common endocrine condition.

Regarding acromegaly, in a study comparing individuals with growth hormone excess to healthy controls (*n* = 8 per group), people with acromegaly had higher mean glucose levels and the percentage of time for blood glucose levels ≥7.8 mmol/L, as well as higher glycaemic variability, although the latter did not reach statistical significance, compared to the control group [95]. In a follow-up analysis of the same cohort [96], individuals with acromegaly had higher 2 h post-OGTT glucose and insulin levels, but lower complexity of the glucose time series index (CGI), a marker of glucose homeostasis calculated by CGM [97], and serum insulin-growth factor 1 levels were negatively associated with CGI [96]. More studies examining the effects of pharmacological and/or surgical treatments for acromegaly on CGM metrics, as well as the role of CGM in predicting disease progression and complications, are warranted.

However, there are no published CGM data on people with other neuroendocrine tumours associated with an impairment of glucose metabolism, such as pheochromocytomas, somatostatinomas, and glucagonomas, as well as primary or secondary hypercortisolism (endogenous Cushing’s syndrome). Importantly, CGM technologies are not licenced for the diagnosis of endocrine or other diseases, and therefore, existing diagnostic strategies should continue until the diagnostic potential of CGM is confirmed.

## 4. Solid Organ Transplantation

People receiving solid organ transplantation have a high risk of developing diabetes mellitus, known as post-transplant diabetes mellitus (PTDM) [13]. PTDM may be caused by both transplant-related factors (e.g., type of organ transplanted, duration, and type of immunosuppression) and traditional risk factors (e.g., obesity, glucose intolerance, or family history of diabetes) and has been associated with reduced graft survival as well as increased risk of cardiovascular disease and all-cause mortality [14,15,16,17]. Hence, screening for PTDM, including pre-transplant assessment and early detection of dysglycaemia in the post-transplant period, is crucial.

The latest international consensus on PTDM [14] recommends OGTT as the preferred test modality for the diagnosis and screening for PTDM, as alternatives, including HbA1c, lack diagnostic sensitivity and correlation with adverse outcomes. In the absence of strong evidence to support its use, CGM was not included in the consensus.

An observational study [98] exploring factors impacting the incidence of PTDM in 60 kidney transplant recipients (KTRs) without diabetes, using 14-day CGM, prior to and post-surgery, in addition to CBG testing during hospitalisation, found that 23.3% of study participants developed PTDM. Major risk factors associated with PTDM included male sex and an increased postoperative percent of time >180 mg/dL (or >10 mmol/L) (%TAR180 mg/dL) detected using CGM. In a prospective longitudinal study, Eleftheriadis et al. [99] assessed the role of 14-day CGM in the prediction of PTDM and IGT as defined by OGTT–derived 2 h plasma glucose or glucose-lowering treatment on day 90, in 46 KTRs without pre-existing diabetes. They found that CGM percent of time >140 mg/dL (or >7.8 mmol/L) (%TAR140 mg/dL) and %TAR180 mg/dL showed excellent in-sample test characteristics regarding PTDM from day 8 onward (days 8–90 receiver operating characteristic [ROC] area under the curve: 0.88–0.99) and regarding PTDM/IGT with the start of maintenance immunosuppression from day 30 onward (days 30–90 ROC area under the curve: 0.88–0.91) (99). Exploratory CGM-derived %TAR140 mg/dL screening thresholds of 31.8% on day 8 and 13.2% on day 30 displayed sensitivities/specificities of 88%/83% for PTDM and 94%/78% for PTDM/IGT on day 90, respectively [99]. Hence, initial data suggest that CGM may have a promising role in PTDM; however, studies with a larger sample size are needed to confirm these preliminary results. Direct head-to-head trials comparing the predictive role of CGM with other established methods of assessing glycaemic risk are also warranted.

## 5. Medications

Several medications have been reported to induce hyperglycaemia or diabetes [100]. In this review, we focus on two categories of medications that are commonly associated with high glucose levels in clinical practice.

*Glucocorticoids (steroids):* The association between glucocorticoid use and the development of new-onset hyperglycemia or diabetes is well established [101]. The development of diabetes or hyperglycaemia in people without diabetes using glucocorticoids is related to the duration, potency, and dose of steroids used. In clinical practice, glucose monitoring in individuals with steroid-induced hyperglycaemia (SIH) is performed with CBG measurements in the inpatient [102] and outpatient setting, which can be painful and traumatising for some people. It also provides limited information to estimate the degree of hyperglycaemia induced by steroid exposure. Using CGM could potentially overcome these challenges and support treatment.

Although it is expected that the blood glucose levels of people without pre-existing diabetes should normalise after stopping glucocorticoids, dysglycaemia may occasionally persist, and such individuals require close monitoring due to the risk of developing diabetes in the future [103]. Currently, an HbA1c measurement after 3 months or fasting glucose or OGTT within 3 months after discontinuing steroids in people without pre-existing diabetes who experienced SIH are suggested for screening for diabetes [102]. The limitation of this strategy is ensuring this testing happens in the current environment of healthcare systems’ overburden, with the potential for delayed diagnosis and treatment. Identifying those individuals at high risk of developing diabetes at an early stage is crucial. Could the use of CGM facilitate this process?

To date, there is limited evidence around the use of CGM in people without pre-existing diabetes who are treated with glucocorticoids. A prospective observational study [104] of CGM in 20 people (50% had no pre-existing diabetes and 35% had prediabetes) with early-stage breast cancer receiving chemotherapy with concurrent corticosteroid supportive care use showed that all individuals developed hyperglycaemia, defined as ≥1 glucose reading ≥140 mg/dL (7.8 mmol/L). A different observational study [105], which aimed to quantify the severity and incidence of SIH by using CGM in 51 inpatients (without or with pre-existing diabetes), who required systemic high-dose steroid therapy for dermatological conditions, found that 22% of people without diabetes and 42% of those with prediabetes developed SIH. These figures highlight the importance of glucose monitoring, especially in inpatients treated with high-dose steroid therapy, where CGM can detect dysglycaemia on admission (even in those with normal initial glucose levels) and identify those individuals at higher risk.

Nevertheless, while costs and requirements for patient and HCPs’ training are important considerations, the use of CGM in people treated with glucocorticoids, who have a high risk of developing diabetes, such as those with metabolic syndrome and insulin resistance or those taking frequent and prolonged courses of steroids, seems to be promising and warrants further research.

*Immune checkpoint inhibitors*: The development of hyperglycaemia in people taking immune checkpoint inhibitors (ICIs) is infrequent, and the incidence varies depending on the receptor(s) targeted [106,107]. ICI-associated hyperglycaemia in those without pre-existing diabetes is largely secondary to autoimmune destruction of the pancreatic beta cells, resulting in irreversible insulin deficiency, which is associated with a high risk of permanent diabetes (ICI-diabetes) and DKA. DKA can be the first presentation of ICI-diabetes [108]. Due to these risks, guidelines recommend routine monitoring of glucose at baseline and with each treatment cycle of ICI during induction, followed by close monitoring for at least 6 months [109]. On the other hand, blood glucose levels are often normal leading up to initial presentation of ICI-diabetes and DKA [110], suggesting that isolated routine monitoring of blood glucose might not be sufficient to predict the rapid development of hyperglycaemia or DKA in these individuals. This raises the question of whether CGM could capture patterns of hyperglycaemia in a timely manner before the onset of DKA. Currently, there is no evidence on the use of CGM in people treated with ICI.

Identifying those at risk of ICI-diabetes will enable HCPs to monitor their glucose control more closely. Could CGM be an effective, useful, and feasible tool for earlier detection of dysglycaemia in high-risk individuals receiving ICIs? This is an area ripe for future research.

## 6. Genetic Syndromes

Several genetic conditions associated with variably penetrant diabetes could represent targets for screening with CGM before the clinical manifestation of diabetes. For example, cystic fibrosis-related diabetes (CFRD) is common in individuals living with cystic fibrosis [111]. While most published studies have examined CGM in individuals with established CFRD, CGM has also been proposed as a screening and/or diagnostic tool for early dysglycaemia in this condition [112]. A systematic review examining the diagnostic accuracy of a single glucose reading >200 m/dL on CGM compared to OGTT for detecting early dysglycaemia in CFRD found no superiority [113]. However, in a validation analysis of 22 individuals without CFRD, Scully et al. applied diagnostic cut-offs derived from a cohort of people with known CFRD to assess the utility of various CGM metrics as a screening tool, and showed that CGM metrics performed better than HbA1c in distinguishing individuals with or without CFRD [114]. More recently, while CGM data did not predict progression to CFRD in individuals without diabetes at baseline, they were associated with changes in weight and body mass index [115]. However, these studies were not designed as screening studies, and this potential application is currently under evaluation in the ongoing ProspeC-F multicentre trial, aiming to assess CGM metrics as predictors of future CFRD and disease progression [116].

Regarding maternally inherited diabetes and deafness (MIDD) in the context of mitochondrial disease, this condition is typically diagnosed in the fourth decade of life, although the range is broad, and progression to insulin dependency is common [117]. However, there are no published data on CGM use prior to diabetes diagnosis, nor on its potential clinical utility in predicting disease occurrence and/or progression.

Conversely, individuals with Maturity-Onset Diabetes of the Young (MODY) 2, a common subtype of MODY associated with inactivating mutations in the *glucokinase* (*GCK*) gene, may only experience mild fasting hyperglycaemia. This group would generally only require treatment with antihyperglycaemic agents during pregnancy. Therefore, this condition might run undiagnosed for several years or be mismanaged as T2DM. However, CGM may be useful in differentiating GCK-MODY from well-controlled T2DM [118], potentially leading to treatment de-escalation or discontinuation in misclassified cases [119]. Given the heritability of MODY, CGM screening in non-affected family members may provide a better understanding of early signs of glucose dysmetabolism before the occurrence of diabetes. However, genetic testing remains the gold standard for MODY diagnosis and should not be replaced by CGM.

With the expansion of multi-omics techniques, several genetic variants have been linked with genetically predicted post-challenge hyperglycaemia or insulin resistance [120,121]. In the first study using CGM in individuals with identified single-nucleotide polymorphisms associated with glucose dysmetabolism, participants with the rs7903146 T-allele in the transcription factor-7-like 2 (*TCF7L2*) gene but without diabetes exhibited higher mean nocturnal glucose levels influenced by body composition, suggesting a potential role in hepatic glucose production [122]. CGM use in people with loss-of-function mutations in the *KCNQ1* gene confirmed the higher rates of prolonged hypoglycaemia compared to matched controls [123]. Similarly, while variants in the ATP-binding cassette transporter sub-family C member 8 (*ABCC8*) gene are classically associated with congenital hyperinsulinism and hypoglycaemia, non-pancreatectomised individuals may develop spontaneous resolution and subsequently diabetes later in life, and CGM could help in their monitoring during transition [124]. Moreover, although CGM technologies are used in individuals with lipodystrophy syndromes in clinical practice [125], there are no published data on their effectiveness as predictive tools of early dysglycaemia or diabetes risk stratification in these cohorts. Similarly, no data exist for CGM’s predictive utility in people with defects in insulin signalling, such as those with mutations in the insulin receptor (*INSR*) and/or protein kinases like *AKT2* genes.

CGM may also hold promise for enhancing care in populations with other genetic syndromes associated directly or indirectly with an increased risk of diabetes. For example, individuals with congenital adrenal hyperplasia (CAH) related to 21-hydroxylase deficiency, an autosomal disorder, are at higher risk of developing insulin resistance and subsequently diabetes [126] due to chronic glucocorticoid use and disrupted diurnal cortisol rhythmicity [127]. Indeed, in a small cohort of 11 children with CAH, CGM use showed an altered glucose pattern with mild to moderate overnight hypoglycaemia paralleled by low cortisol levels [128], suggesting CGM’s potential utility in guiding dose adjustments in those individuals. However, there is a paucity of data in adult populations with CAH, and it is conceivable that CGM technologies may provide valuable insights to support personalised care in this group.

CGM may be useful for phenotyping or monitoring treatment de-escalation in misclassified cases of genetic syndromes, but its role should be considered exploratory. Genetic testing and conventional methods such as OGTT remain the gold standard. Future family-based longitudinal studies could help quantify the heritability of CGM phenotypes and their relationship to incident diabetes.

## 7. Precision Approaches

The wider availability of large datasets and bioinformatics methodologies has spurred a quest for greater personalisation in medical care. CGM is an important tool in delivering individualised care to people with existing diabetes. It increases flexibility and allows for adjustments based on day-to-day situations, such as meal ingestion, exercise, other personal or lifestyle parameters. Furthermore, it is increasingly common for healthy people to self-fund CGM to observe how their lifestyle impacts GV. Additionally, several companies have made CGM commercially available, and CGM data from the general population have informed analyses of glycaemic patterns and individualιzed responses to meals, with preliminary studies on precision nutrition emerging in people at risk of diabetes [129,130]. Beyond wellness and lifestyle applications, CGM has been used by elite athletes to monitor glucose fluctuations during their training sessions and optimise their performance [131]. However, its potential advantages in precision prevention attempts for individuals at risk of diabetes are less explored.

Adherence to dietary guidelines has been problematic for various reasons, with less than 0.1% of the UK population following all nine Eatwell Guide recommendations and just over 30% meeting at least five recommendations [132]. While the existing evidence is currently limited, using CGM feedback could potentially motivate individuals to adhere to personalised, healthy lifestyle changes [133]. Indeed, although it failed to meet its primary outcome in low-density lipoprotein cholesterol improvements, the ZOE Measuring Efficacy Through Outcomes of Diet (METHOD) study, an 18-week RCT of a personalised nutrition intervention incorporating CGM surveillance, showed significant improvements in exploratory outcomes of dietary, other biochemical, and cardiometabolic health parameters in individuals without diabetes [134]. Ben-Yacov et al. demonstrated an improvement in CGM-assessed mean time glucose levels >140 mg/dL (7.8 mmol/L) in individuals with prediabetes who followed a personalised, postprandial-targeting diet compared to those who were assigned a Mediterranean diet [135]. Therefore, it is possible that CGM could emerge as a core tool in future precision strategies, with the added benefit of capturing diurnal data in real-world settings. However, Hengist et al. recently showed high intraindividual variability of glucose responses to duplicate meals derived from CGM metrics in individuals without diabetes [136], suggesting that such data may not be reliable in guiding personalised nutrition strategies yet [136,137]. Further research is required. Integrating CGM with electronic health record and genomic or other omics data, as well as information generated from wearables tracking physical activity and sleep, could further enhance CGM’s utility, and may yield more detailed prediction models of lifestyle patterns and glucose outcomes [48] compared to the current approach of one-snapshot, clinic-based investigations.

Using these approaches in tandem may also deepen our understanding of early glucose dysmetabolism and individualised diabetes risk, including intra- and inter-person and/or ethnic variability. This could, in turn, potentially inform the design and implementation of targeted strategies for diabetes prevention in high-risk individuals with similar characteristics. However, the cost-effectiveness and efficacy of these approaches must be confirmed in future longitudinal studies and RCTs before being implemented on a large scale. Until then, clinicians are encouraged to use existing tools for risk assessment and prioritising care.

## 8. Future Directions and Conclusions

Currently, there are several unanswered questions around the use of CGM in people at high risk of developing diabetes. In this context, most HCPs are awaiting evidence-based information before suggesting wider implementation of CGM to these populations. It should be noted that CGM-derived data are not validated for the diagnosis of diabetes, and potential challenges include the lack of evidence and consensus for defining abnormal CGM values in people without diabetes, as well as guidance on further management. Also, faulty or inaccurate CGM sensors may provide inaccurate readings, leading to inappropriate actions and related anxiety [138]. This highlights the importance for a CGM user to understand the indications and limitations of this technology. While most studies have used a 10–14-day monitoring period, the optimal duration, frequency, and cost-effectiveness of CGM in this context are unclear. Long-term feasibility in healthcare systems remains a challenge and is an important consideration for future research. It is also unclear whether a single two-week trial of a CGM device in a free-living environment would be adequate or longer than required to provide representative and reproducible data and to capture inter- and/or intraindividual variability in people at risk of developing diabetes. Also, heterogeneity between devices, calibration methods, and regional availability limits the comparability of CGM-derived data across countries, underscoring the need for device-agnostic validation before wider adoption. This is particularly important if CGM technologies are to be employed at scale in the design of remote, population-level screening strategies. While CGM provides an alternative to clinic-based assessments for the diagnosis and/or monitoring of individuals at risk, potentially alleviating the burdened healthcare systems worldwide, the cost and accessibility, data overload, and anxiety, as well as user adherence, need to be taken into consideration before scaling up CGM use. HCPs should distinguish between hypothesis-generating associations, often from small observational studies, and clinically validated evidence supported by RCTs or consensus guidelines.

Although limited data have been reported to date on the benefits of CGM for people at high risk of developing diabetes, a high interest in defining the role of this technology in these populations has begun. CGM offers a promising approach to identify and support the management of glycaemic abnormalities in people at high risk of diabetes. At present, evidence supports research use and selective adjunctive clinical monitoring in defined high-risk contexts. Diagnostic use will require validated thresholds, using devices that meet required accuracy and precision thresholds and demonstrate improved patient-important outcomes and cost-effectiveness before widespread adoption. Table 2 summarises the CGM metrics reported across studies, the cut-off thresholds applied (where available), and their predictive value or clinical associations.

Further research is needed to clarify the indications, drawbacks, and feasibility of CGM use in people at high risk of diabetes, as well as identify those groups who could benefit most from this technology. Future research priorities should focus on the following: (1) defining standardised CGM thresholds for early diabetes diagnosis, identifying glucose patterns that signal disease onset; (2) validating these thresholds across populations in large, prospective cohorts, ensuring they are linked to clinically meaningful outcomes; (3) assessing cost-effectiveness and feasibility of implementing CGM in routine preventive care; and (4) integrating CGM with multi-omics and wearable data to enable precision risk prediction.

## Figures and Tables

**Table 1:** Potential future applications and limitations of CGM across high-risk groups for dysglycaemia and diabetes. Abbreviations: CGM, Continuous Glucose Monitoring; MASLD, Metabolic dysfunction-Associated Steatotic Liver Disease; PCOS, Polycystic Ovarian Syndrome; MODY, Maturity-Onset Diabetes of the Young; CFRD, Cystic Fibrosis-Related Diabetes; OGTT, Oral Glucose Tolerance Test.

High-Risk Group	Rationale for CGM Use	Potential Future Applications	Limitations/Considerations
Pre-symptomatic type 1 diabetes (islet autoantibody positivity)	Early detection of glycaemic excursions before overt diabetes Higher acceptability in children and families [8,9]	Risk stratification Monitoring in prevention trials	Lack of evidence for the following: Cost-effectiveness Psychological burden Clear diagnostic thresholds
Metabolic dysfunction (Prediabetes, early type 2 diabetes, and altered body composition)	Capturing postprandial hyperglycaemia and glucose variability missed by commonly used glucose metrics Enhancing adherence to lifestyle modifications through real-time biofeedback	Replacing clinic-based glycaemic measurements (e.g., OGTT and HbA1c) Adherence monitoring Precision interventions, including precision nutrition	Uncertain cut-offs Resource implications
Gestational diabetes mellitus	Rapid glycaemic changes Maternal/foetal outcomes linked to high glucose variability [10,11]	Early screening and risk stratification tool (13–14 gestational weeks) [12] Reducing neonatal complications Empowering self-management and lifestyle changes	Cost-effectiveness Absence of specific CGM targets
MASLD PCOS Acromegaly	Commonly associated with insulin resistance and dysglycaemia	Identifying postprandial hyperglycaemia Guiding dietary interventions Early detection of progression Monitoring effects of pharmacological/surgical interventions	Lack of validated CGM thresholds Limited evidence base Small sample sizes
Solid organ transplantation	High risk of post-transplant diabetes associated with reduced graft survival and mortality [13,14,15,16,17]	Early detection of dysglycaemia	Small sample sizes Absence of direct comparative trials with gold standard methods
Steroid/Immune checkpoint inhibitors therapy	Therapy-induced hyperglycaemia is often unpredictable	Monitoring glycaemic excursions remotely Reduce healthcare overburden guiding prophylactic/early interventions	Costs and requirements for patient and healthcare professionals’ training
Genetic syndromes (e.g., MODY, CFRD, etc.)	Unique pathophysiology of dysglycaemia Early identification of high glycaemic variability	Understanding glycaemic patterns Phenotyping or monitoring treatment de-escalation in misclassified cases of genetic syndromes Research applications, including family-based heritability studies	Rare diseases, limited clinical trial data Genetic testing and conventional methods such as OGTT remain the gold standard

**Table 2:** Summary of CGM metrics, cut-off thresholds, and predictive values/clinical associations across high-risk populations. Abbreviations: CGM, Continuous Glucose Monitoring; PTDM, Post-Transplant Diabetes Mellitus; IGT, Impaired Glucose Tolerance; CFRD, Cystic Fibrosis-Related Diabetes; AUROC, Area Under the Receiver Operating Characteristic Curve.

High-Risk Group	CGM Metric(s)	Cut-Off Thresholds	Predictive Values
Pre-symptomatic type 1 diabetes (islet autoantibody positivity) [8]	Interstitial fluid glucose levels	Glucose ≥ 140 mg/dL (≥7.8 mmol/L) for >10% per day	88% sensitivity and 91% specificity for diabetes prediction
	Glycaemic variability	20 mg/dL (1.1 mmol/L) standard deviation	81% sensitivity and 81% specificity for diabetes prediction
	Mean amplitude of glucose excursion	37 mg/dL (2.1 mmol/L)	69% sensitivity and 91% specificity for diabetes prediction
Prediabetes [37]	Functional assessment of glucose homeostasis (FLAG)	Prediabetes defined as per American Diabetes Association criteria	86% sensitivity and 71–78% specificity for prediabetes
Gestational diabetes mellitus [66]	Second trimester and gestational week 13–14 percent time > 140 mg/dL (>7.8 mmol/L)	Percent time > 140 mg/dL (>7.8 mmol/L)	AUROC 0.81 when using second trimester percent time > 140 mg/dL (>7.8 mmol/L) AUROC 0.74 when using percent time > 140 mg/dL (>7.8 mmol/L) as early as 13–14 gestational weeks
PTDM [99]	Percent time > 140 mg/dL (>7.8 mmol/L)	Exploratory screening thresholds of 31.8% on day 8 and 13.2% on day 30	AUROC for days 8–90 post-transplant: 0.88–0.99
CFRD [114]	Percent time > 140 mg/dL (>7.8 mmol/L) and/or 180 mg/dL (10 mmol/L)	17.5% time > 140 mg/dL (>7.8 mmol/L); 3.4% time > 180 mg/dL (>10 mmol/L)	sensitivities of 87% (for 140 mg/dL; 7.8 mmol/L) and 90% (for 180 mg/dL; 10 mmol/L) specificities of 95% AUROC: 94%

## Data Availability

Not applicable.

## Abbreviations

ABCC8	ATP-binding cassette transporter sub-family C member 8
CAH	congenital adrenal hyperplasia
CBG	capillary blood glucose
CFRD	cystic fibrosis-related diabetes
CGI	glucose time series index
CGM	continuous glucose monitoring
DKA	diabetic ketoacidosis
GCK	glucokinase
GDM	gestational diabetes mellitus
GV	glucose variability
GRADE	grading of recommendations, assessment, development, and evaluations
HbA1c	haemoglobin A1c
HCPs	healthcare professionals
ICIs	immune checkpoint inhibitors
IGT	impaired glucose tolerance
*INSR*	insulin receptor gene
KTRs	kidney transplant recipients
MASLD	metabolic dysfunction-associated steatotic liver disease
MIDD	maternally inherited diabetes and deafness
MODD	mean of daily differences
MODY	maturity-onset diabetes of the young
OGTT	oral glucose tolerance test
PCOS	polycystic ovarian syndrome
PTDM	post-transplant diabetes mellitus
RCTs	randomised controlled trials
ROC	receiver operating characteristic
RYGB	Roux-en-Y gastric bypass
SD	standard deviation
SIH	steroid-induced hyperglycaemia
T1DM	type 1 diabetes mellitus
T2DM	type 2 diabetes mellitus
*TCF7L2*	transcription factor-7-like 2 gene
TIR	time-in-range
